# Virtual Screening for Reactive Natural Products and Their Probable Artifacts of Solvolysis and Oxidation

**DOI:** 10.3390/biom10111486

**Published:** 2020-10-27

**Authors:** Tingjun Xu, Weiming Chen, Junhong Zhou, Jingfang Dai, Yingyong Li, Yingli Zhao

**Affiliations:** Shanghai Institute of Organic Chemistry, Chinese Academy of Sciences, 345 LingLing Road, Shanghai 200032, China; chenwm@sioc.ac.cn (W.C.); zhoujh8@sioc.ac.cn (J.Z.); daijf@sioc.ac.cn (J.D.); liyingyong@sioc.ac.cn (Y.L.); zhaoyl@sioc.ac.cn (Y.Z.)

**Keywords:** natural product, artifact, virtual screening, deep learning

## Abstract

Chemically unstable natural products are prone to show their reactivity in the procedures of extraction, purification, or identification and turn into contaminants as so-called “artifacts”. However, identification of artifacts requires considerable investments in technical equipment, time, and human resources. For revealing these reactive natural products and their artifacts by computational approaches, we set up a virtual screening system to seek cases in a biochemical database. The screening system is based on deep learning models of predicting the two main classifications of conversion reactions from natural products to artifacts, namely solvolysis and oxidation. A set of result data was reviewed for checking validity of the screening system, and we screened out a batch of reactive natural products and their probable artifacts. This work provides some insights into the formations of natural product artifacts, and the result data may act as warnings regarding the improper handling of biological matrixes in multicomponent extraction.

## 1. Introduction

The large diversity of natural products from one biological source leads to difficulties in multicomponent extraction. In the black box of exploring undiscovered natural products, options of handling, storage, or analysis of a biological matrix are often empirical or semiempirical. Under such circumstances, some chemically unstable molecules are prone to show their reactivity in the procedures of extracting natural products and turn into contaminants as so-called “artifacts” [1]. The artifacts arise from the products of non-enzymatic reactions during the process of extraction or purification [2,3,4]. In most cases, these reactions are between natural products and solvents or chromatography media [1,2,3,4]. On the other hand, oxidation of natural products when exposed to air or light is also common [3,4]. The revealing of artifacts usually occurs in chemical experiments on a case-by-case basis, and extensive artifact search and discovery may rely on computational approaches of virtual screening or data-mining. Few extensive searches have been done because of technical limitations and deficiency of specialized data resources in natural product chemistry [5,6].

New computational approaches for explorations in chemistry were booming in the last decade, particularly in the application of machine learning (ML) [7,8]. Researchers applied various algorithms of ML such as deep learning (DL) to design novel molecules, predict chemical properties, or plan reaction paths [9,10,11,12,13,14,15]. Neural networks were applied to reaction prediction in some studies by ranking electron sources and sinks or generating reaction fingerprints [10,14]. Further applications regarded chemical reactions as transformations [13]. The transformations can be considered as translations from reactants to products, and the “language” being translated is the structural representation of molecules. In the mechanism insights into the formations of natural product artifacts, we can regard the formations as transformations from natural products to artifacts, and we can predict the transformations by computational approaches. With the advantage of using ML, we might have an efficient and convenient approach to identify specific classifications of reactions, instead of building complex models. Therefore, we pursued an exploration of seeking more cases of chemically reactive natural products and their probable artifacts that have not been documented. We set up a deep-learning-based virtual screening system for discovering these extraordinary natural products in a specialized data set.

## 2. Materials and Methods

According to investigation into studies that reported artifacts, the transformations of natural products to artifacts are reactions in specific classifications (e.g., solvolysis and oxidation) [1,2,3,4]. Identifying these reactions means that seeking out reactive natural products (reactants of these reactions) and their artifacts (products of these reactions) and using computational approaches may be a better approach than the use of chemical experiments in consideration of investments in technical equipment, time, and human resources. We herein take advantage of virtual screening, which is applicable for the task of searching for and discovering exceptional molecules in a database, and use virtual screening to target reactive natural products. In the theoretical base of the virtual screening used in this study, the core idea is to determine specific classifications of reactions that cause artifacts. We realized this conception by using ML to predict probable products of these reactions. If a natural molecule and its predicted product are derived from the same biological source, we have a theoretical clue to suspect that the molecule is a reactive natural product and the predicted product is its artifact. Therefore, we can seek for potential cases by checking for the existence of these reactions in a specialized data set. The specialized data set used in this study is a biochemical database (http://www.organchem.csdb.cn/scdb/NPBS) [16]. In this data resource, the relational data (relationship between a specific biological source and all the natural products derived from it reported by various studies) includes sufficient natural products from various biological sources. An example of a set of relational data listed in Table 1 describes 10 natural products from *Thalictrum delavayi.* More detailed example data are included in the Supplementary Materials.

**Figure 1:**
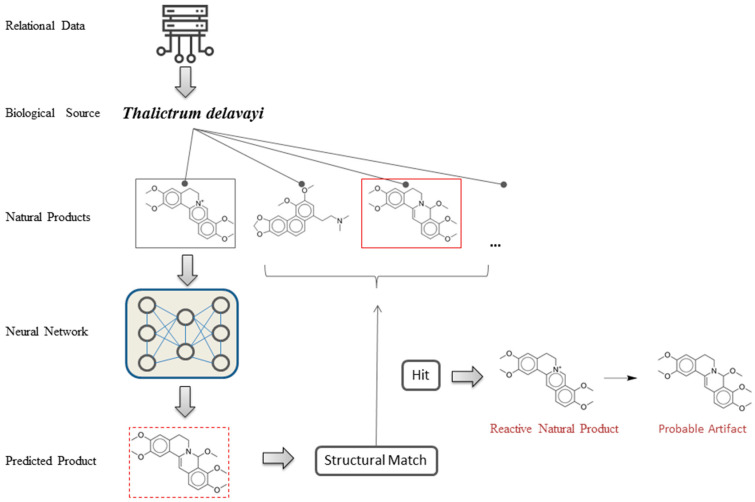
Illustration of the virtual screening system for discovering reactive natural products and their probable artifacts [17,18].

We assumed that a small fraction of the natural products were reactive in the process of extraction and there were corresponding artifacts extracted from the same biological source. In that case, a reactive natural product and its artifact would probably coexist in a set of relational data (reported by one or more studies). The reactive natural product and its artifact would form a set of reaction data of a reactant and a product, and the specific reaction could be predicted by our trained models. According to the features of the data set, we designed a virtual screening strategy as follows (also as shown in Figure 1):

1. Take a set of relational data (a specific biological source and all the natural products derived from it);
2. Take one of the natural product molecules in this relational data set;
3. Predict its solvolysis and oxidation products by neural network models;
4. If predictions of the models are successful (or partially successful), match the predicted products with the other natural product molecules from the same biological source;
5. If a predicted product matches one of the other natural product molecules, label the natural product and the predicted product as a potential case;
6. Go through steps 2–5 with all the other molecules in the same relational data set;
7. Go through steps 1–6 with all the other relational data sets and screen out all the potential cases in the data set.

In Step 4 of the procedure, the success of predictions is judged based on the validity of the SMILES strings for molecular structure generated by the models, and the judgment is made by RDKit. In the vast majority of successful cases, only one model among the models we built generated a valid SMILES string and could be described as “partially successful”.

Available information on transformations from natural products to artifacts is rare and implicit in the literature. A set of preliminary data was extracted from studies where such information was available [1,2,3,4]. The preliminary data set is paired with molecules as natural products transform into artifacts. With the knowledge of these transformations from the preliminary data set, we expanded analogous transformations to common chemical reactions in specific classifications from a reaction database [19]. The reactions were classified based on the two main causes of artifacts: solvolysis and oxidation [1,2,3,4]. The reactions of solvolysis are compounds reacted with or in solvents. Solvents or media such as methanol, ethanol, acetone, dichloromethane, chloroform, and water are commonly used in natural product extraction [1,2,3]. The reactions of oxidation are compounds transformed into oxides with the effect of air, light, or heat [4]. The data set was made up of reactants (except solvents, catalysts, or other participants) and products (except by-products) from the reaction data set. We used these data as the training data set for our deep-learning-based approach. For normalization of the data, the structural representations of reactants and products are canonicalized SMILES strings using an implicit representation of hydrogen atoms [10,20]. The processed data set is included in the Supplementary Materials.

**Figure 2:**
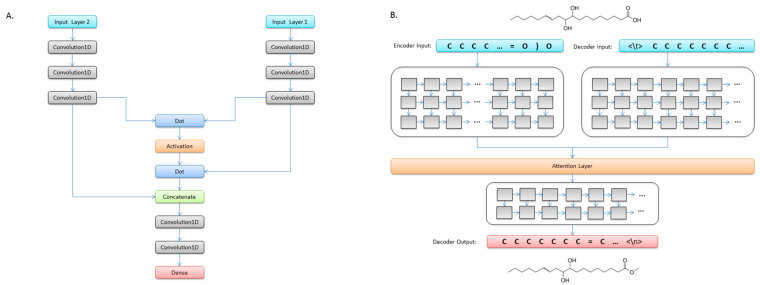
(**A**) Architecture of the neural networks for predicting the reactions of natural products to artifacts. (**B**) Illustration of the convolutional neural network (CNN)-based neural networks in training mode.

Convolutional neural networks (CNNs) are deep learning architectures well suited to the translation of variable-length sequences such as text sentences [21,22]; herein, we extrapolate such techniques to SMILES strings of molecular structures. In the theoretical base of the used virtual screening, the core idea is to determine the specific classifications of reactions that cause artifacts, and we realized this conception by using CNN models to predict the probable products of these reactions. Thus, we applied an attention-based CNN model for predicting the reactions of natural products to artifacts [23]. We dealt with the transformations of SMILES strings as language translation, taking the reactants as source sentences and the products as target sentences. The neural network model conceptually consists of four elements: an encoder of three one-dimensional CNN layers that encodes the input character sequence, a decoder of three one-dimensional CNN layers that turns the target sequences into the same sequence but offset by one timestep in the future, attention mechanism layers that take the outputs of the encoder and decoder, and a decoder of two one-dimensional CNN layers that decodes the output character sequence, as shown in Figure 2. The input SMILES strings of natural products are transformed into embedding sets of vectors. The number of vectors equals the number of unique characters in all input SMILES strings and is provided as an input to the encoder–decoder model with attention mechanism. The output SMILES strings are reversed from predicted sequences by re-embedding.

The models were trained on seven classifications of reaction from the training data set: solvolysis of methanol, ethanol, acetone, dichloromethane, chloroform, and water and oxidation. The training data for CNN models were from the reaction data set described above. We split the data set for cross-validation at random, 80% for training set and 20% for validation set. We took the reactants of the reaction data as source data, taking the products as target data. The parameters of the neural networks were chosen according to the performances on the validating set (key hyperparameters of the best-performing CNN models are listed in Table 2), and other parameters remained unchanged as default settings of the used neural network architecture [21,22,23,24,25,26]. We obtained the top percentages of correctly predicted products in seven classes, as listed in Table 3. We used the best-performing models to predict the potential transformations of natural products to artifacts. The models were implemented in Python 3.7 using Keras 2.3 and TensorFlow backend [24,25,26]. The Python code for generating the neural network models is included in the Supplementary Materials. We applied RDKit in Python for generating SMILES strings and processing molecular structures [27].

## 3. Results and Discussion

We first obtained a set of natural products and successfully predicted products from the seven CNN models. The first result data set consists of molecular information of the natural products and predicted products, along with the specific CNN model that generated the SMILES strings of predicted products, that would form a group of reactive natural products and their probable artifacts with biological source information in our virtual screening system according to the theoretical base of this work. Results from the virtual screening system were reviewed to check the validity of our approach and seek positive data. We eventually screened out 118 cases of reactive natural products and their probable artifacts from the biochemical database. The result data set consists of reactive natural products, probable artifacts, biological sources, probable causes, and references (data sources for biological sources and natural products). The complete result data sets and the trained model files of this work are included in Supplementary Materials. Some of the cases are listed in following figures as discussions of typical examples we found, and the original images of these figures are also included in Supplementary Materials as ChemDraw files.

**Figure 3:**
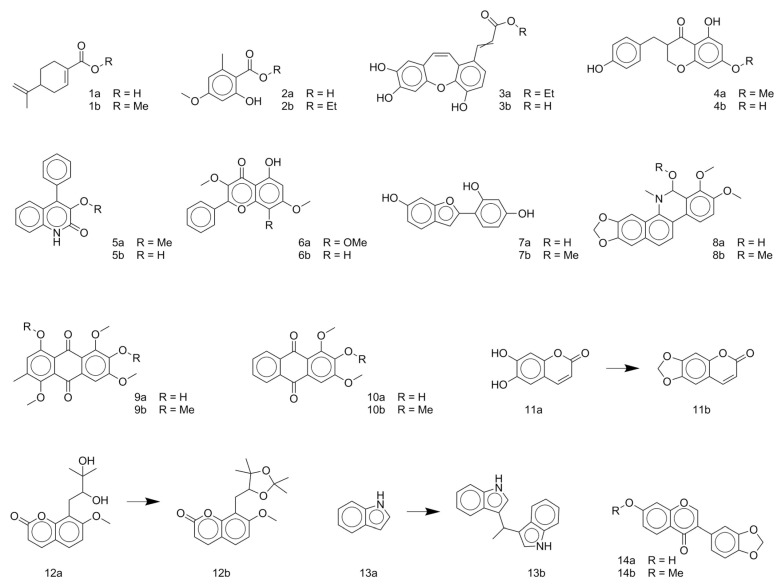
Some typical cases of reactive natural products and their probable artifacts caused by solvolysis in the result data set.

As observed from the result data set, natural products with carboxylic groups may react with the common solvents of alcohols (e.g., methanol and ethanol) (Figure 3). Perilla acid (**1a**) derived from *Pectis elongata* may form methyl perillate (**1b**) [28,29]. 4-*O*-Methylorsellinic acid (**2a**) derived from *Usnea longissima* may form its Et ester (**2b**) [30]. Although ethoxy groups are rare in nature, not all the esterified carboxylic acids can be seen as artifacts [1]. Tournefolic acid B Et ester (**3a**) may hydrolyze to tournefolic acid B (**3b**) when isolated from the stems of *Tournefortia sarmentosa* [31]. The homoisoflavonoids derived from *Ledebouria graminifolia* may count in 5-hydroxy-3-(4-hydroxybenzyl)-7-methoxychroman-4-one (**4a**) and 5,7-dihydroxy-3-(4-hydroxybenzyl)chroman-4-one (**4b**), and the latter may be a hydrolysate of the former [32]. Similarly, viridicatin (**5b**) derived from *verrucosum* var. *cyclopium* may be the hydrolysate of 3-methoxy-4-phenylquinolin-2(1H)-one (**5a**) [33]. Bioassay-guided fractionation of *Cryptocarya chinensis* may cause the hydrolysis of 5-hydroxy-3,7,8-trimethoxyflavone (**6a**) and produce 5-hydroxy-3,7-dimethoxyflavone (**6b**) [34]. Erythbidin D (**7b**) isolated from the roots of *Erythrina* × *bidwillii* may be the product of methylation from erythbidin E (**7a**) [35]. Similarly, 6-hydroxy-5,6-dihydrochelerythrine (**8a**) may form angoline (**8b**) when isolated from *Zanthoxylum nitidum* using chromatography [36]. More cases of methylation are included in the result data set. For example, pseudobaptigenin (**14a**) isolated from *Sophora japonica L.* may form 7-O-methylpseudobaptigenin (**14b**) [37,38]. Natural products containing quinone substructures may react with nucleophilic solvents (e.g., methanol). 4,6-Dihydroxy-1,5,7-trimethoxy-2-methylanthraquinone (**9a**) derived from *Chamaecrista greggii* may form its methide (**9b**) [39]. 1,3-Dimethoxy-2-hydroxyanthraquinone (**10a**) also may form its methide (**10b**) [40]. The use of dichloromethane in chromatography may cause the transformation from 6,7-dihydroxycoumarin (**11a**) to ayapin (**11b**) [41]. Acetone may react with natural product meranzin (**12a**) and transform it into a probable artifact (**12b**) when isolated from dried fruitlets of *Citrus grandis* [42]. The EtOAc extract of the whole culture medium of *Vibrio parahaemolyticus* may cause an unexpected reaction, which can turn 1H-indole (**13a**) into a probable artifact vibrindole A (**13b**) [43].

**Figure 4:**
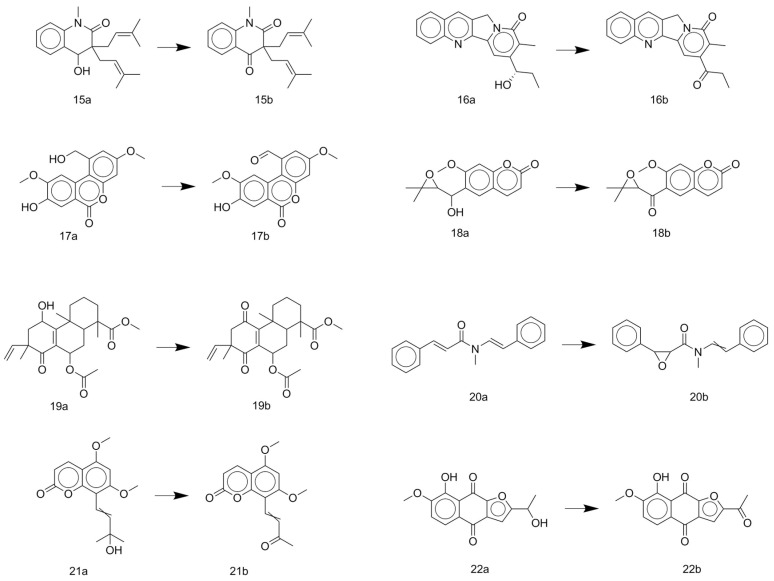
Some typical cases of reactive natural products and their probable artifacts caused by oxidation in the result data set.

The oxidation of natural products to produce artifacts is also common, as observed from the result data set, especially in the cases of natural products containing benzylic alcohol substructures (Figure 4). Fractionation of trunk wood and roots of *Esenbeckia almawillia* may cause an oxide of 3,3-diisopentenyl-*N*-methyl-2,4-quinoldione (**15b**) [44]. Nothapodytines A (**16b**) may be also an oxide when isolated from the stems of *Nothapodytes foetida* [45]. Other cases of oxidation from benzylic alcohols are herpetolide A (**17a**), which may form herpetolide B (**17b**) when extracted from the seeds of *Herpetospermum caudigerum* [46], and lophopterol (**18a**), which may form hopeyhopin (**18b**) when isolated from the root of *Citrus paradisi* [47]. The oxidation of hydroquinones to quinones is also found in the result data set. Metabolites of *Lycopus europaeus* may include an oxide of methyl 7α-acetoxy-11,14-dioxo-8,15-isopimaradien-18-oate (**19b**) [48]. Ether extract of the seeds of *Clausena lansium* may cause the oxidation of lansiumamide C (**20a**) [49]. Coumarin (**21a**) may have an oxidative cleavage reaction when isolated from the roots of *Toddalia asiatica*, forming a probable artifact (**21b**) [50]. Another case of an artifact caused by oxidation is the fractionation of the stem bark of *Tabebuia ochracea* ssp. *neochrysanta*, which may lead to an oxide of naphtho[2,3-b]furan-4,9-diones (**22b**) [51].

## 4. Conclusions

The architecture of the neural networks (CNNs) is well suited to the translation of variable-length sequences, such as text sentences and, as used in this work, the SMILES strings of molecular structures. However there may be practical limitations for wider chemical spaces, seeing that the CNNs are more applicable for translation of short sentences [52]. In the case of large molecules or synthetic reactions, the length of SMILES strings and the complexity of the data space have restricted such techniques, preventing them from being used in wider applications.

Although the transformations (or reactions) from natural products to artifacts predicted by neural networks are restrained to the superficial level, the predictions lacking information related to chemical mechanism, and the virtual screening strategy relies on relational data and assumptions. The potential reactivity of molecules determined just by inspection of data may be without chemical proof, and there are some products of transformations that may not actually be natural product artifacts. For example, there are some oxidized variants of natural products that are either secondary metabolites themselves or represent the action of further metabolism in the producing organism in detoxifying a compound or preparing it for excretion; therefore, it may be arbitrary to suggest that the oxides are all artifacts. However, the results of this work provide some insights into the formations of natural product artifacts.

Although artifacts are unexpected contaminants, exploiting those transformations may inspire the synthesis of new chemical diversity. The result data with biological source information can act as warnings regarding the improper handling of biological matrixes in multicomponent extraction. This work is far from authenticating the artifacts experimentally, and some of the transformations seem impossible, but we hope the relationships and information obtained from the specialized data set provide some knowledge of reactive natural products and their artifacts in natural product chemistry.

## Figures and Tables

**Table 1:** Example of a set of relational data: natural products from *Thalictrum delavayi.*

No.	Biological Source	Natural Product
1	*Thalictrum delavayi*	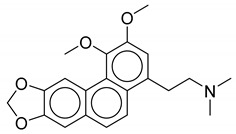
2	*Thalictrum delavayi*	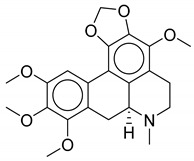
3	*Thalictrum delavayi*	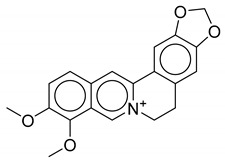
4	*Thalictrum delavayi*	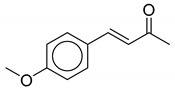
5	*Thalictrum delavayi*	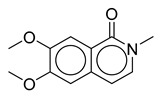
6	*Thalictrum delavayi*	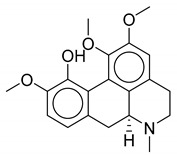
7	*Thalictrum delavayi*	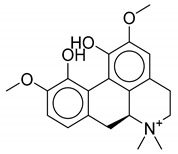
8	*Thalictrum delavayi*	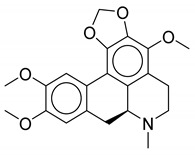
9	*Thalictrum delavayi*	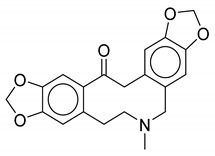
10	*Thalictrum delavayi*	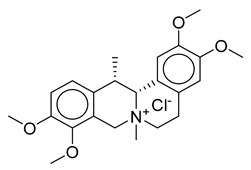

**Table 2:** Key hyperparameters of the best-performing CNN models.

Class of CNN Model	Batch Size	Epoch	Latent Dimensionality of Encoding Space	Latent Dimensionality of Decoding Space	Optimizer
Solvolysis of methanol	64	100	256	64	Adam
Solvolysis of ethanol	64	500	256	64	Adam
Solvolysis of acetone	64	100	256	64	Adam
Solvolysis of dichloromethane	64	500	256	64	Adam
Solvolysis of chloroform	64	1000	256	64	Adam
Solvolysis of water	64	500	256	64	Adam
Oxidation	64	500	256	64	Adam

**Table 3:** Performance of the used CNN models on validation data set.

Class of CNN Model	Success	Concordance	Accuracy
Solvolysis of methanol	88.21%	0.93	75.72%
Solvolysis of ethanol	86.80%	0.87	78.27%
Solvolysis of acetone	98.18%	0.97	87.91%
Solvolysis of dichloromethane	95.00%	0.97	89.64%
Solvolysis of chloroform	88.64%	0.96	85.23%
Solvolysis of water	82.33%	0.86	70.40%
Oxidation	86.86%	0.85	71.07%

Success: percentage of valid SMILES strings for molecular structure generated by the models; Concordance: average sequence match ratio of target and predicted SMILES strings (0 = totally different, 1 = exact match); Accuracy: percentage of chemical structure identification (same InchiKey) between target and predicted SMILES strings.

## Supplementary Materials

The following are available online at https://www.mdpi.com/2218-273X/10/11/1486/s1: Example of a set of relational data: natural products from *Thalictrum delavayi*. The data set for training and validation. The python code for generating and implementing the neural network models. Figures S1–S7: Training accuracy figures of the used models, Figures S8–S14: Scatter plots figures of the used models on validation data set, the result data sets, the ChemDraw files of original images. Some typical cases of reactive natural products and their probable artifacts caused by solvolysis and oxidation in the result data set.
