# Supplementary material for: Virtual Screening for Reactive Natural Products and Their Probable Artifacts of Solvolysis and Oxidation
Source: Biomolecules. 2020 Oct 27;10(11):1486. doi: 10.3390/biom10111486 (PMC7692644; doi:10.3390/biom10111486)
Supplement: Supplementary file 1 [file biomolecules-10-01486-s001.zip › 5.Training accuracy figures of the CNN models (Figures S1-S7).docx]

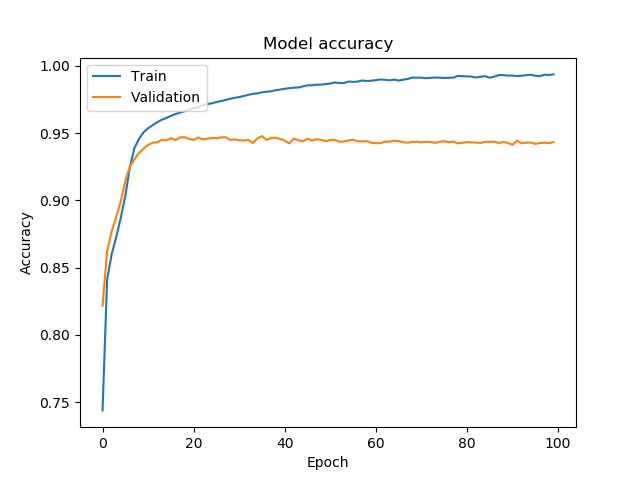


Figure S1. Solvolysis of methanol


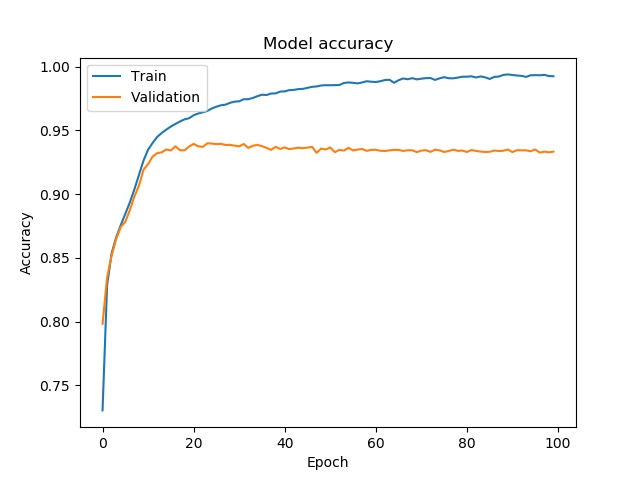


Figure S2. Solvolysis of ethanol


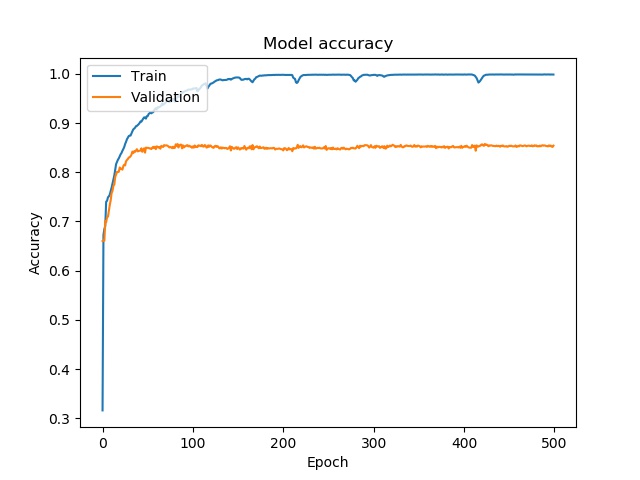


Figure S3. Solvolysis of acetone
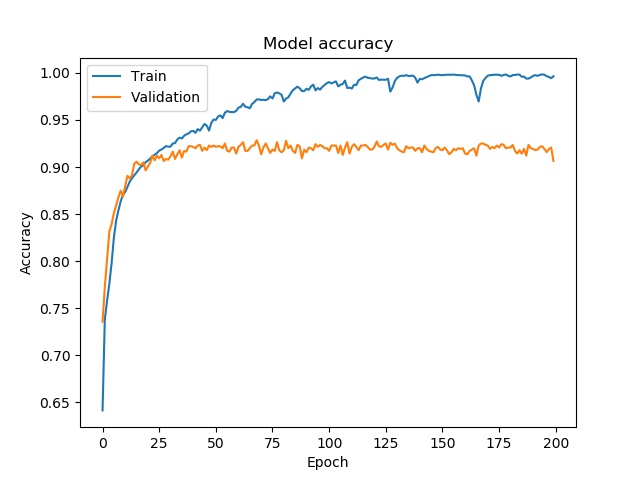


Figure S4. Solvolysis of dichloromethane


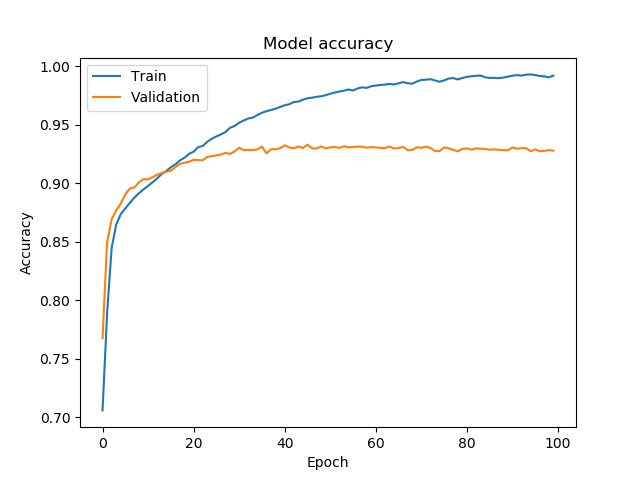


Figure S5. Solvolysis of chloroform


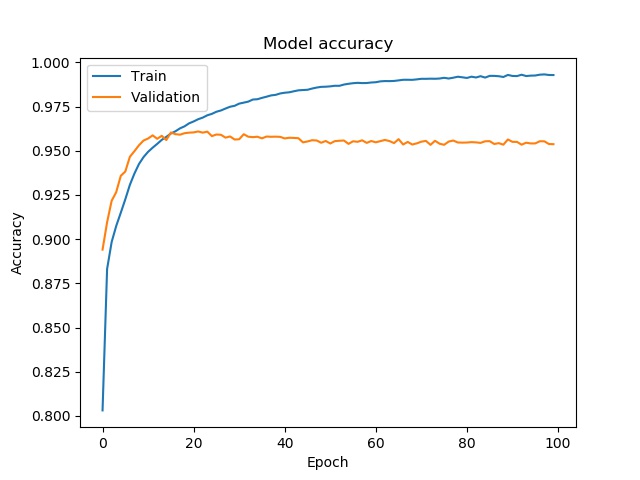


Figure S6. Solvolysis of water


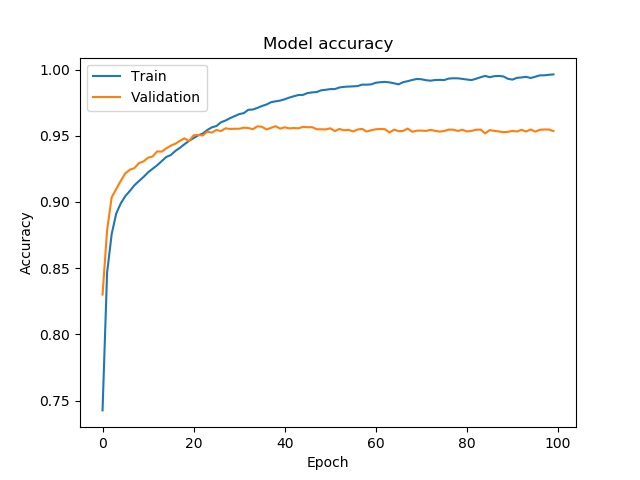


Figure S7. Oxidation
