# Supplementary material for: Virtual Screening for Reactive Natural Products and Their Probable Artifacts of Solvolysis and Oxidation
Source: Biomolecules. 2020 Oct 27;10(11):1486. doi: 10.3390/biom10111486 (PMC7692644; doi:10.3390/biom10111486)
Supplement: Supplementary file 1 [file biomolecules-10-01486-s001.zip › 6.Scatter plots figures of the used models on validation data set (Figures S8-14).docx]

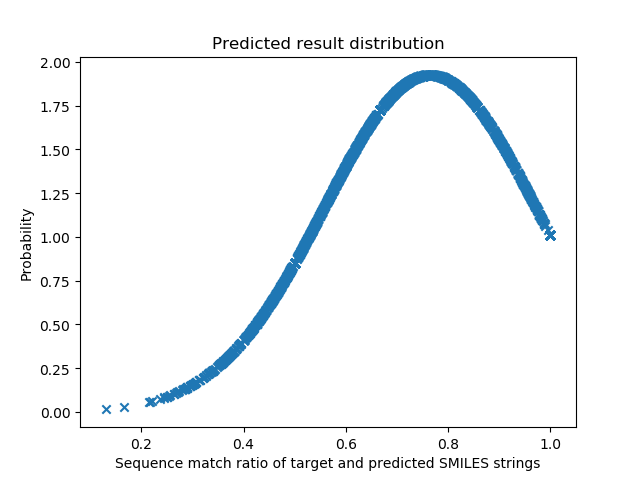


Figure S8. Solvolysis of methanol


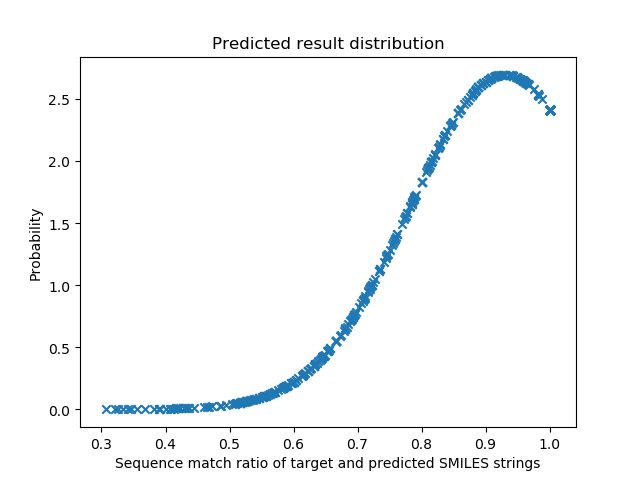


Figure S9. Solvolysis of ethanol


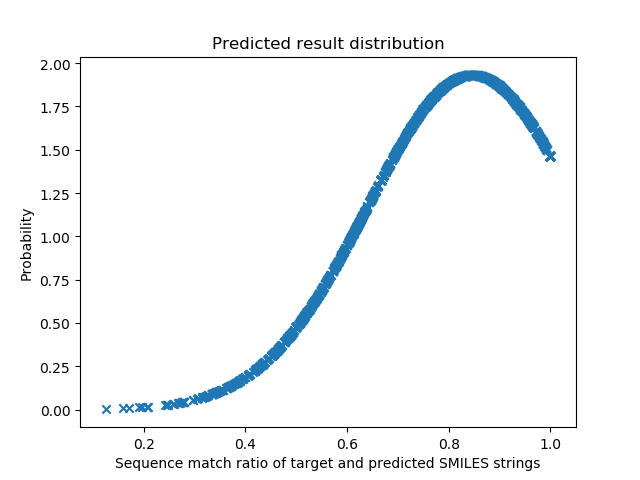


Figure S10. Solvolysis of acetone


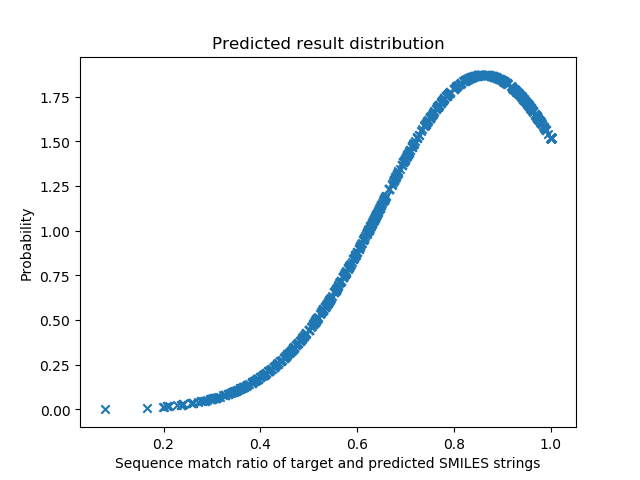


Figure S11. Solvolysis of dichloromethane


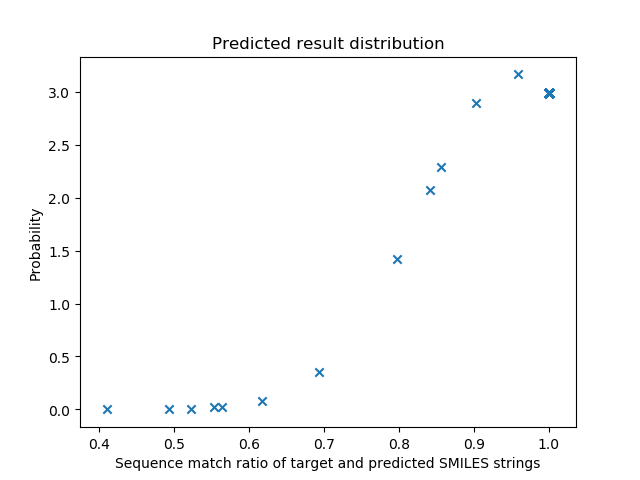


Figure S12. Solvolysis of chloroform


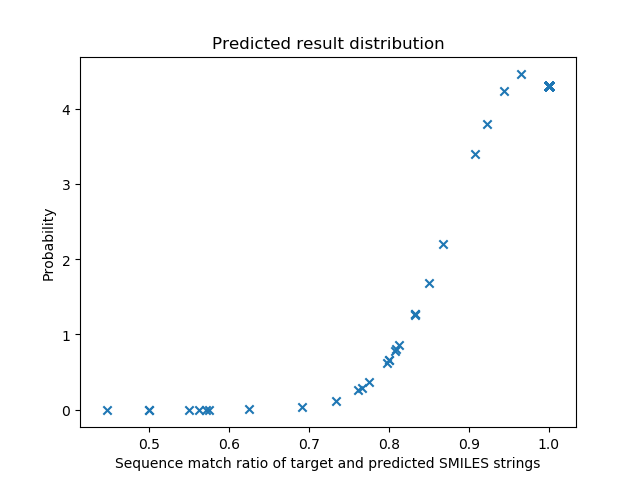


Figure S13. Solvolysis of water


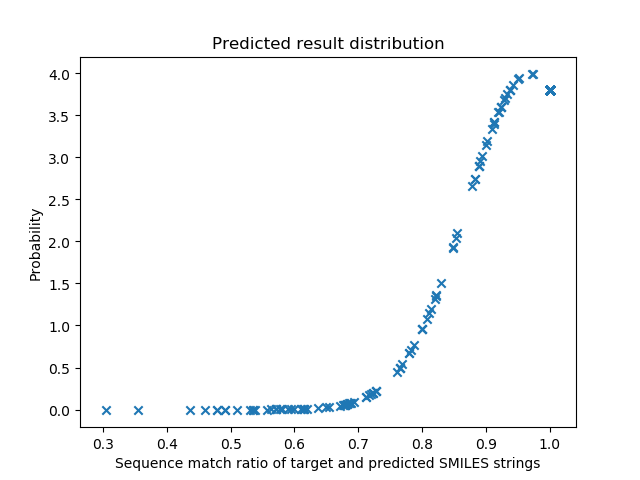


Figure S14. Oxidation
