# Supplementary material for: Virtual Screening for Reactive Natural Products and Their Probable Artifacts of Solvolysis and Oxidation
Source: Biomolecules. 2020 Oct 27;10(11):1486. doi: 10.3390/biom10111486 (PMC7692644; doi:10.3390/biom10111486)
Supplement: Supplementary file 1 [file biomolecules-10-01486-s001.zip › 8.Result data set.pdf]

The result data set of "Virtual Screening for Reactive Natural Products and Their Probable Artifacts of Solvolysis and Oxidation"

Tingjun Xu et al.

Reactive natural product	Probable artifact	Probable cause	Biological source	References
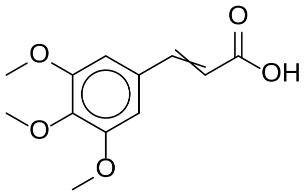	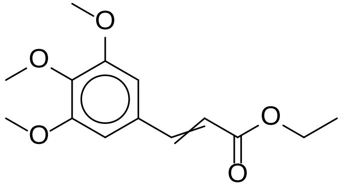	Solvolysis	*Polygala tenuifolia*	Zhongguo Zhongyao Zazhi, 33(11), 1278-1280<br>Zhongguo Yaowu Huaxue Zazhi, 15(1), 35-38
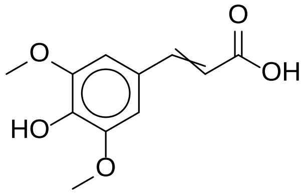	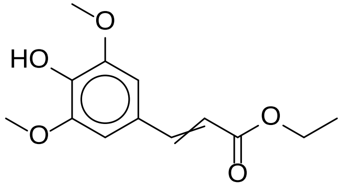	Solvolysis	*Descurainia sophia*	Journal of Asian Natural Products Research, 7(6), 853-856<br>Shenyang Yaoke Daxue Xuebao, 20(6), 419-421
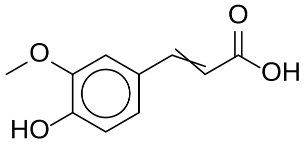	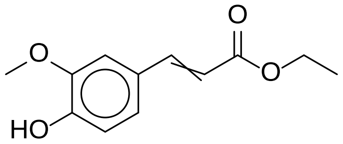	Solvolysis	*Curcuma longa*	Food Chemistry, 119(3), 974-980<br>Tianran Chanwu Yanjiu Yu Kaifa, 15(2), 98-100
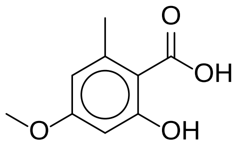	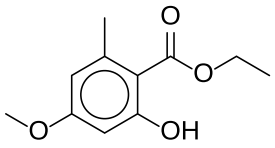	Solvolysis	*Usnea longissima*	Phytochemistry, 26(12), 3181-3185<br>Yunnan Zhiwu Yanjiu, 8(4), 483-488

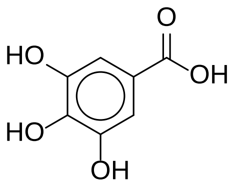	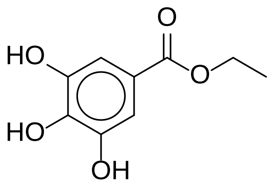	Solvolysis	*Dimocarpus longan*	Food Chemistry, 116(2), 433-436<br>Huazhong Keji Daxue Xuebao, Yixueban, 38(4), 524-526
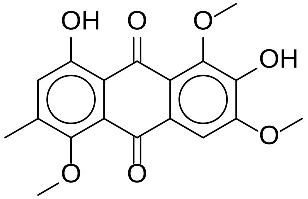	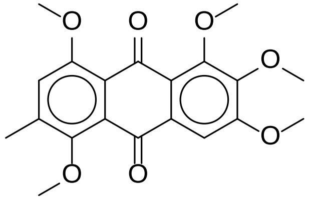	Solvolysis	*Chamaecrista greggii*	Phytochemistry, 37(3), 837-845
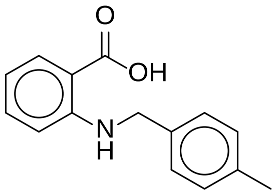	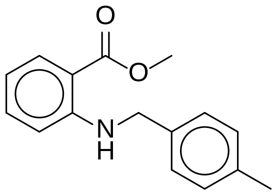	Solvolysis	*Onosma hispida*	Chemical & Pharmaceutical Bulletin, 53(8), 907-910
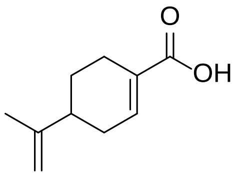	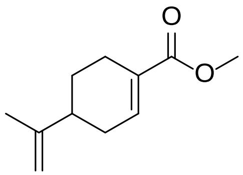	Solvolysis	*Pectis elongata*	Flavour and Fragrance Journal, 20(5), 462-464
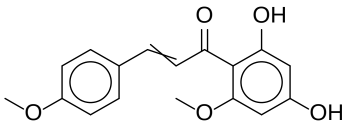	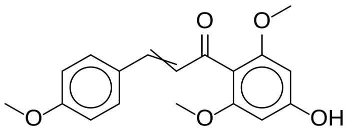	Solvolysis	*Vitex leptobotrys*	Phytochemistry, 49(8), 2603-2605

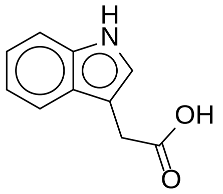	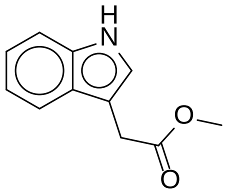	Solvolysis	*Isatis tinctoria*	Xiandai Zhongyao Yanjiu Yu Shijian, 23(2), 54-56<br>Planta Med., 61(1), 95-96
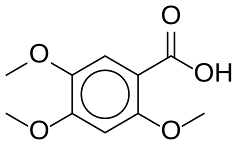	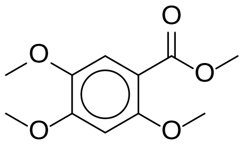	Solvolysis	*Calanthe arisanensis*	Bioorganic & Medicinal Chemistry Letters, 18(15), 4275-4277
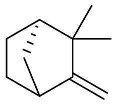	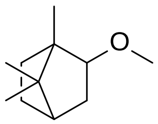	Oxidation	*Artemisia annua*	Flavour Fragrance J., 12(4), 241-246
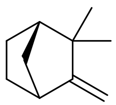	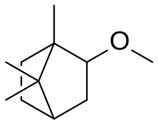	Solvolysis	*Artemisia annua*	Flavour Fragrance J., 12(4), 241-246
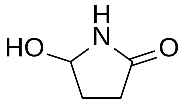	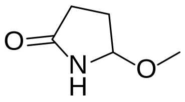	Solvolysis	*Bistorta manshuriensis*	Natural Product Sciences, 15(4), 234-240
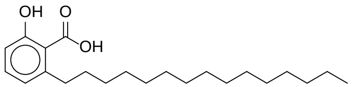	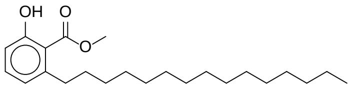	Solvolysis	*Ozoroa insignis*	Journal of the Brazilian Chemical Society, 17(3), 527-532
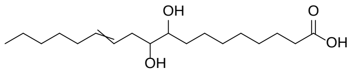	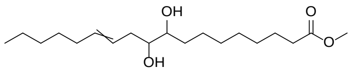	Solvolysis	*Cucurbita pepo*	Agric. Biol. Chem., 41(1), 175-180

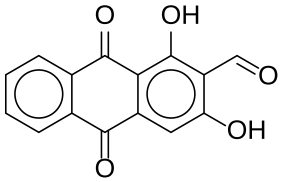	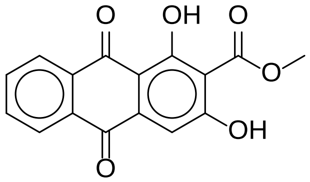	Solvolysis	*Rubia wallichiana*	Chemical & Pharmaceutical Bulletin, 51(8), 948-950
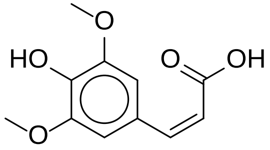	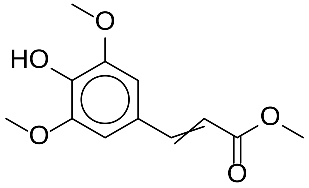	Solvolysis	*Brassica napus*	Journal of Agricultural and Food Chemistry, 62(13), 2935–2945
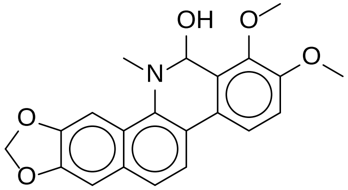	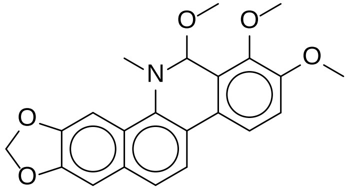	Solvolysis	*Zanthoxylum nitidum*	Zhongcaoyao, 40(4), 538-540<br>Chemistry & Biodiversity, 3(9), 990-995
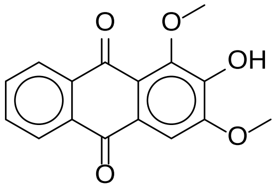	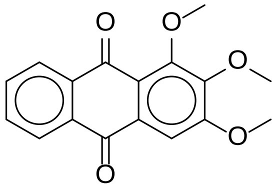	Solvolysis	*galium sinaicum*	Phytochemistry, Volume Date 1992, 31(1), 355-356
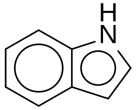	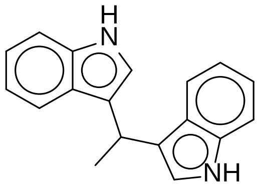	Solvolysis	*Vibrio parahaemolyticus*	J. Nat. Prod., 57(11), 1587-1590

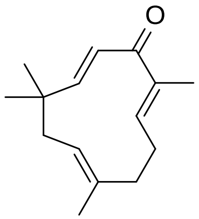	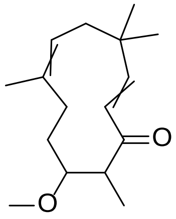	Oxidation	*Zingiber zerumbet*	Journal of Essential Oil Research, 15(3), 202-205<br>J. Essent. Oil Res., 5(1), 55-59
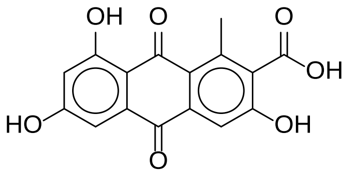	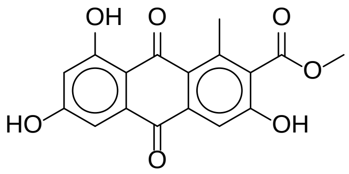	Solvolysis	*Gladiolus gandavensis*	Journal of Asian Natural Products Research, 7(3), 197-204
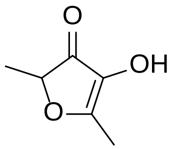	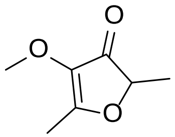	Solvolysis	*Mangifera indica*	J. Agric. Food Chem., 46(3), 1094-1100<br>Phytochemistry, 24(10), 2313-2316<br>Food Science and Technology Research, 7(3), 200-206<br>Food Chemistry, 114(1), 363-372
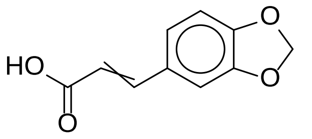	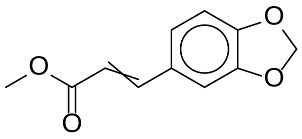	Solvolysis	*Justicia diffusa var. prostrata*	Journal of Asian Natural Products Research, 2(4), 289-300
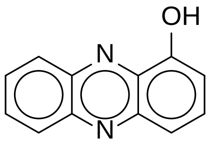	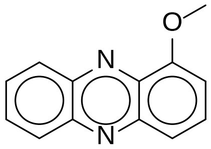	Solvolysis	*Streptomyces sp.SCSIO1042*	Zhongguo Haiyang Yaowu, 33(1), 45-52

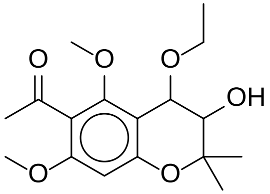	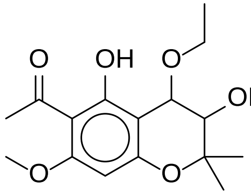	Solvolysis	*Evodia lepta*	Phytochemistry, Volume Date 1998, 47(1), 101-104<br>Zhiwu Xuebao, 39(7), 670-674
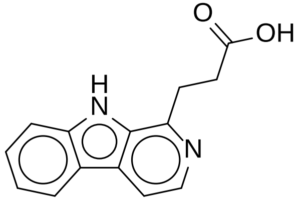	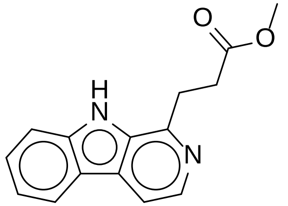	Solvolysis	*Picrasma quassioides*	Chem. Pharm. Bull., 32(9), 3579-3583<br>Huaxue Xuebao, 42(7), 679-683
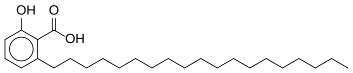	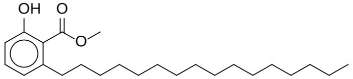	Solvolysis	*Ozoroa insignis*	Biochemical Systematics and Ecology, 37(2), 116-119<br>Journal of the Brazilian Chemical Society, 17(3), 527-532
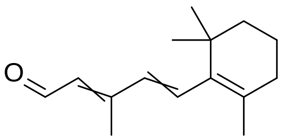	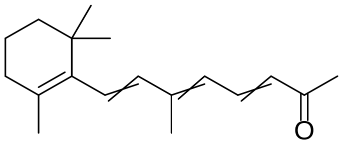	Solvolysis	*Equisetum fluviatile*	Journal of Essential Oil Research, 20(5), 437-441
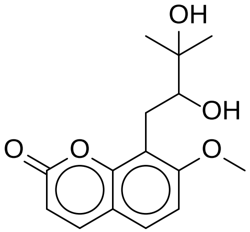	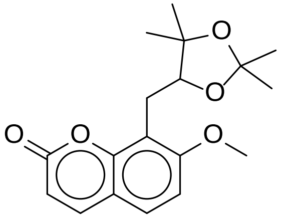	Solvolysis	*Citrus grandis*	Shenyang Yaoke Daxue Xuebao, 17(4), 253-255<br>Zhongcaoyao, 36(3), 341-343

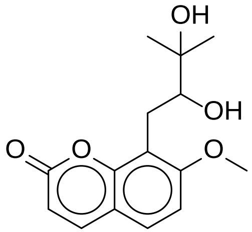	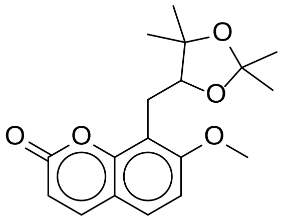	Solvolysis	*Citrus grandis*	Zhongcaoyao, 36(3), 341-343<br>Zhongguo Zhongyao Zazhi, 26(11), 764-765<br>Shenyang Yaoke Daxue Xuebao, 17(4), 253-255
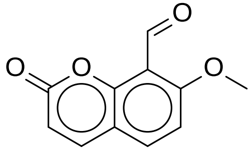	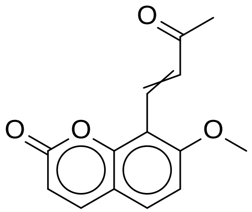	Solvolysis	*Murraya exotica*	Chem. Pharm. Bull., 35(10), 4277-4285
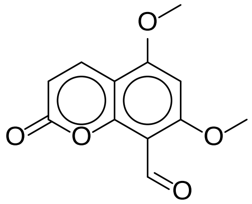	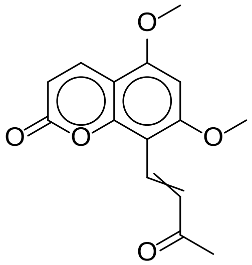	Solvolysis	*Toddalia asiatica*	Yakugaku Zasshi, 111(7), 365-375
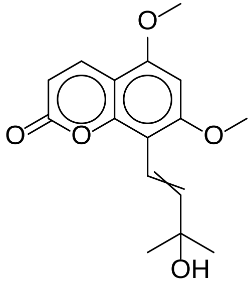	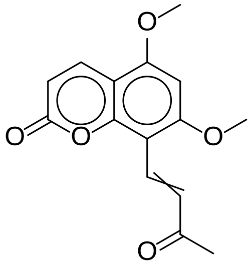	Oxidation	*Toddalia asiatica*	Yakugaku Zasshi, 111(7), 376-385<br>Fitoterapia, 71(6), 636-640

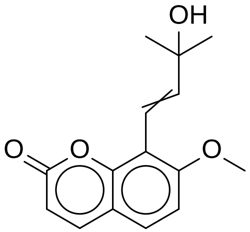	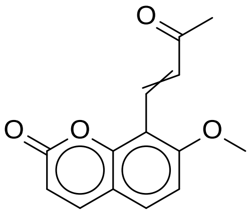	Oxidation	*Murraya exotica*	Chem. Pharm. Bull., 35(10), 4277-4285
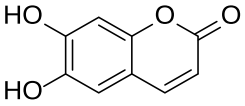	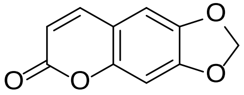	Solvolysis	*Protium javanicum*	Journal of Chemical Ecology, 36(7), 720-726
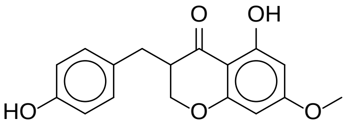	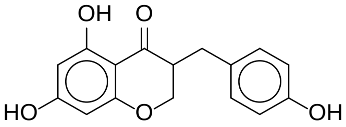	Solvolysis	*Ledebouria graminifolia*	Phytochemistry (Elsevier), 62(5), 797-804
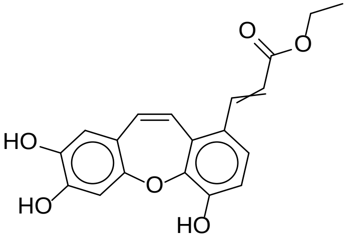	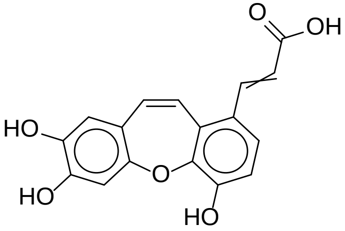	Solvolysis	*Tournefortia sarmentosa*	Journal of Natural Products, 65(5), 745-747
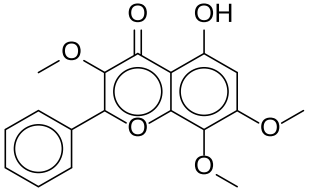	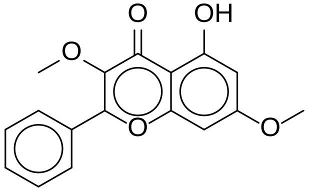	Solvolysis	*Cryptocarya chinensis*	Journal of Natural Products, 73(9), 1470-1475

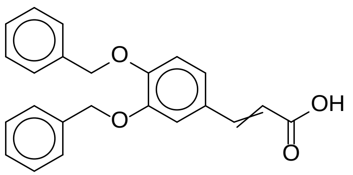	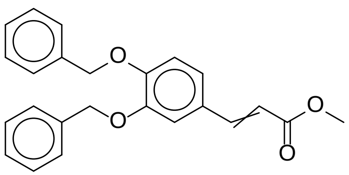	Solvolysis	*Justicia diffusa var. prostrata*	Journal of Asian Natural Products Research, 2(4), 289-300
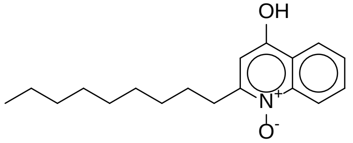	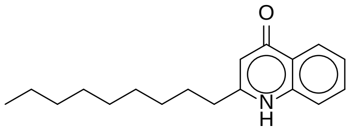	Oxidation	*Pseudomonas aeruginosa*	Monatsh. Chem., 110(4), 947-53
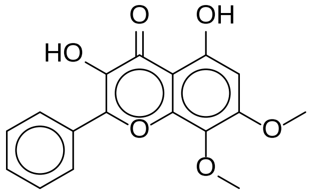	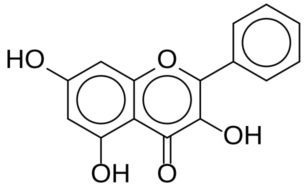	Solvolysis	*Woodsia scopulina*	Phytochemistry, 49(3), 859-862
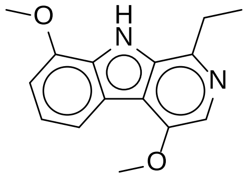	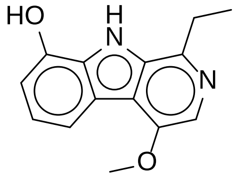	Solvolysis	*Picrasma quassioides*	Zhongcaoyao, 38(6), 807-810
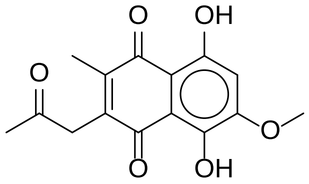	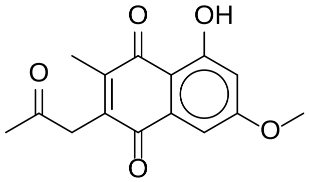	Solvolysis	*Fusarium sp*	Chemistry of Natural Compounds, 50(6), 1103-1105

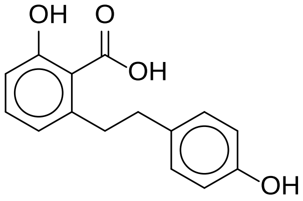	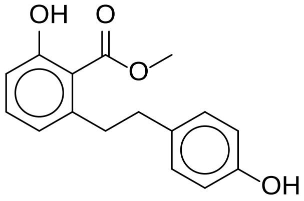	Solvolysis	*Ricciocarpos natans*	Planta Med., 56(5), 444-5<br>Phytochemistry, 31(11), 3981-3
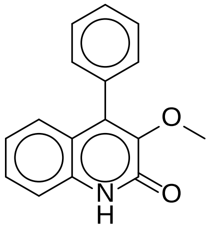	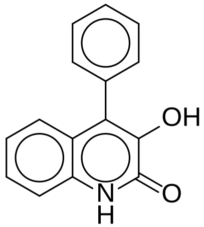	Solvolysis	*penicillium verrucosum var. cyclopium*	J. Nat. Prod., 51(1), 66-73
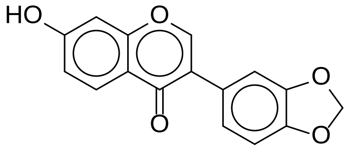	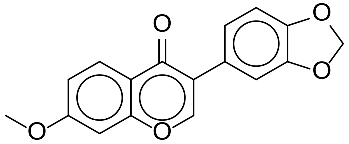	Solvolysis	*Sophora japonica*	Journal of Asian Natural Products Research, 4(1), 1-5<br>Zhongcaoyao, 33(1), 20-21
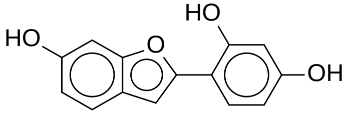	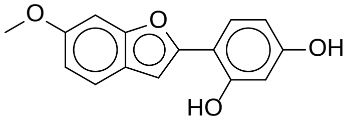	Solvolysis	*Erythrina ?bidwillii*	Heterocycles, 71(8), 1779-1785
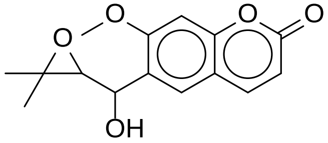	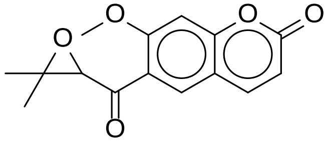	Oxidation	*Citrus paradisi Macf.*	Chem. Pharm. Bull., 44(4), 804-809

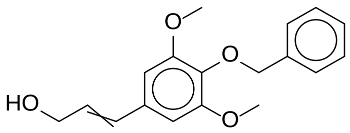	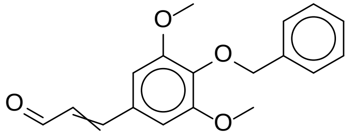	Oxidation	*Ligularia nelumbifolia*	Journal of Natural Products, 65(6), 902-908
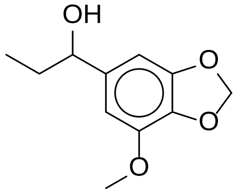	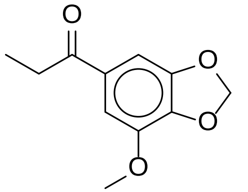	Oxidation	*Ferula latipinna*	J. Nat. Prod., 51(6), 1140-1147
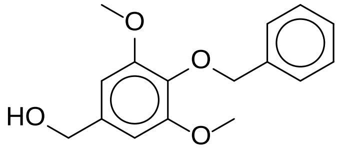	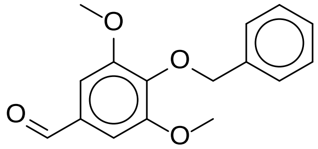	Oxidation	*Ligularia nelumbifolia*	Journal of Natural Products, 65(6), 902-908
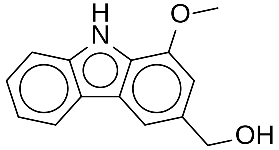	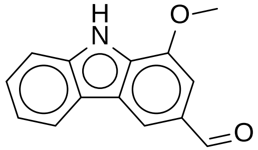	Oxidation	*Murraya koenigii*	Chem. Pharm. Bull., 41(12), 2096-20100
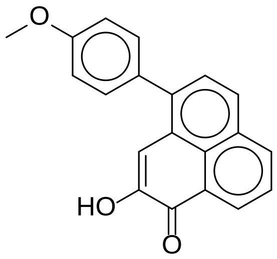	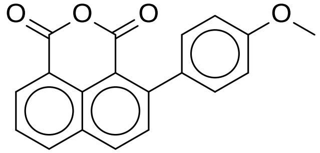	Oxidation	*Musa acuminata*	Biosci., Biotechnol., Biochem., 62(1), 95-101

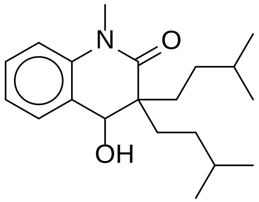	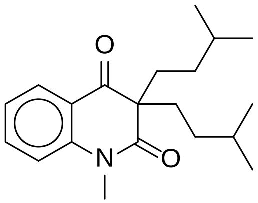	Oxidation	*Esenbeckia almawillia*	Natural Product Communications, 1(4), 313-318
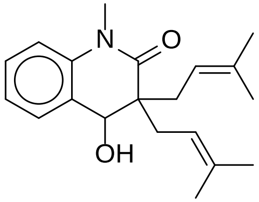	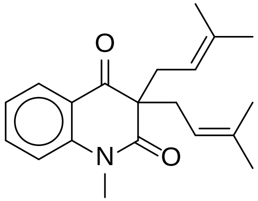	Oxidation	*Esenbeckia almawillia*	Biochemical Systematics and Ecology, 32(9), 817-821
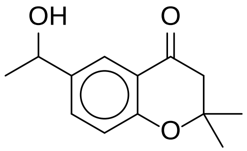	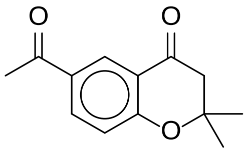	Oxidation	*Artemisia campestris subsp. glutinosa*	Phytochemistry, 22(11), 2587-2589
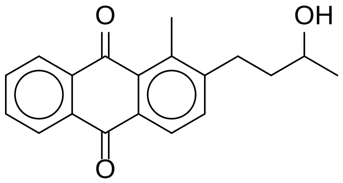	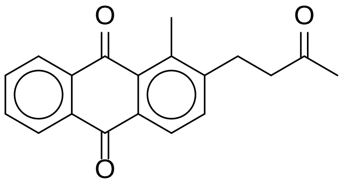	Oxidation	*Stereospermum personatum*	Journal of Natural Products, 68(11), 1615-1621
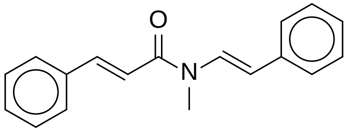	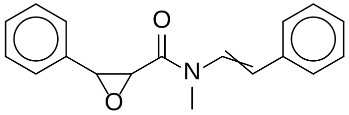	Oxidation	*Clausena lansium*	Phytochemistry, 28(2), 621-622<br>Chin. Pharm. J. (Taipei), 48(5), 367-373

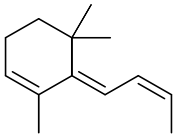	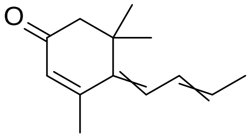	Oxidation	*Averrhoa carambola*	Guoshu Xuebao, 25(1), 119-121<br>Flavour Fragrance J., 7(4), 179-185
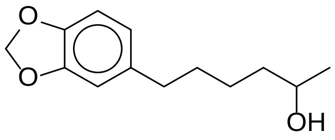	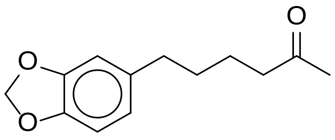	Oxidation	*Ruta angustifolia*	Phytochemistry, 25(9), 2209-2210<br>J. Essent. Oil Res., 3(5), 355-357
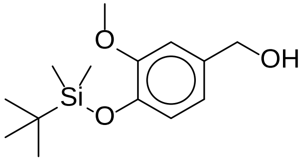	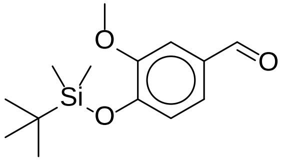	Oxidation	*Stereospermum personatum*	Journal of Natural Products, 68(11), 1615-1621
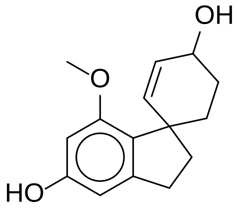	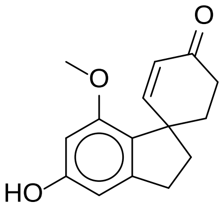	Oxidation	*Cannabis sativa*	Pharm. Acta Helv., 59(8), 216-224<br>J. Chem. Soc., Perkin Trans. 1, (7), 1455-1466
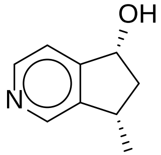	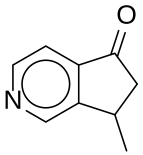	Oxidation	*Coelospermum billardieri*	J. Nat. Prod., 51(5), 829-835

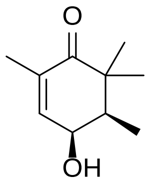	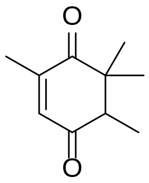	Oxidation	*Iris germanica*	Helv. Chim. Acta, 72(6), 1400-1415
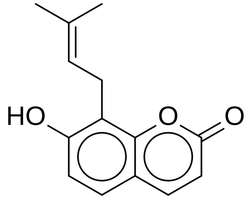	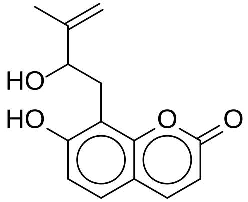	Oxidation	*Citrus paradisi Macf.*	Chem. Pharm. Bull., 44(4), 804-809
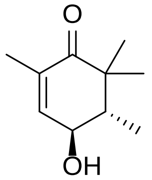	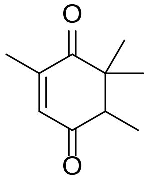	Oxidation	*Iris germanica*	Helv. Chim. Acta, 72(6), 1400-1415
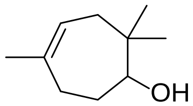	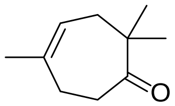	Oxidation	*Cupressus sempervirens*	Parfums, Cosmet., Aromes, 20, 33-6, 39-41
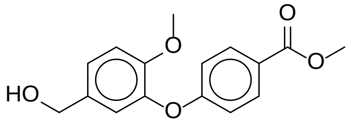	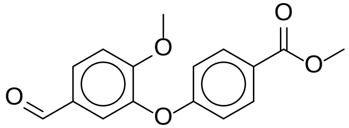	Oxidation	*Aristolochia elegans*	Bioorganic & Medicinal Chemistry, 12(2), 439-446<br>Journal of Natural Products, 65(11), 1522-1525
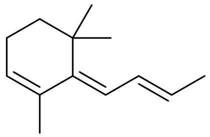	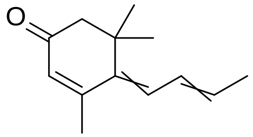	Oxidation	*Averrhoa carambola*	Guoshu Xuebao, 25(1), 119-121<br>Flavour Fragrance J., 7(4), 179-185

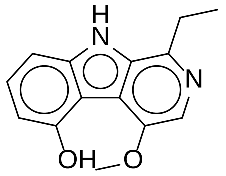	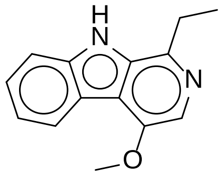	Oxidation	*Picrasma javanica*	Aust. J. Chem., 40(9), 1527-1536<br>Chem. Pharm. Bull., 41(12), 2101-2105
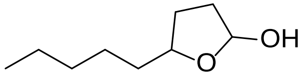	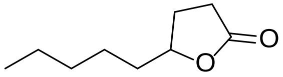	Oxidation	*Citrullus vulgaris*	Agric. Biol. Chem., 49(11), 3145-3150
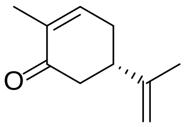	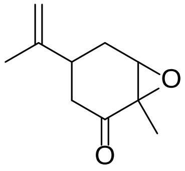	Oxidation	*Mentha spicata*	Harbin Shangye Daxue Xuebao, Ziran Kexueban, 19(1), 72-74, 92
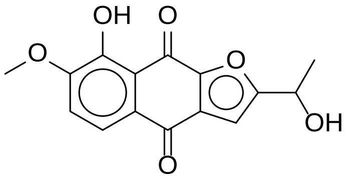	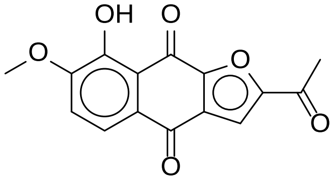	Oxidation	*Tabebuia ochracea neochrysanta*	J. Nat. Prod., 59(4), 423-424
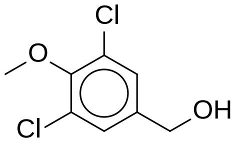	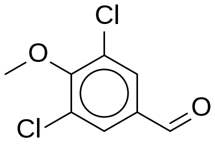	Oxidation	*Pholiota destruens*	Nat. Prod. Lett., 5(3), 171-174
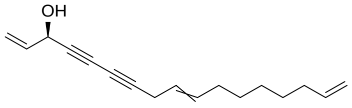	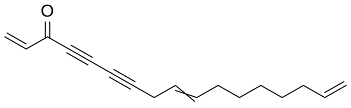	Oxidation	*Artemisia capillaris*	Phytochemistry, 21(8), 2009-2011

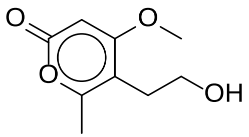	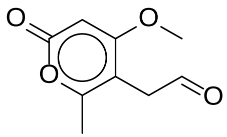	Oxidation	*Macrophoma commelinae*	Chem. Pharm. Bull., 31(10), 3781-3784<br>Chem. Pharm. Bull., 36(4), 1328-1335
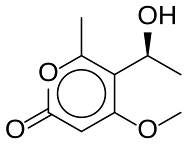	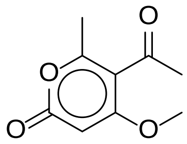	Oxidation	*Macrophoma commelinae*	Chem. Pharm. Bull., 31(10), 3781-3784<br>Chem. Pharm. Bull., 36(4), 1328-1335
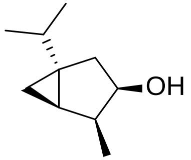	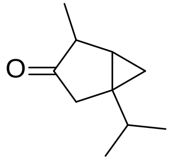	Oxidation	*Chrysanthemum indicum*	Chemistry & Biodiversity, 7(12), 2951-2962<br>Anjisuan He Shengwu Ziyuan, 31(4), 69-75
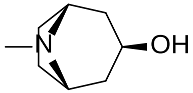	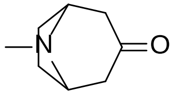	Oxidation	*Datura stramonium*	Biochemical Systematics and Ecology, 33(10), 1017-1029
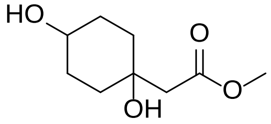	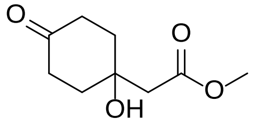	Oxidation	*Senecio scandens*	Biochemical Systematics and Ecology, 35(12), 901-904
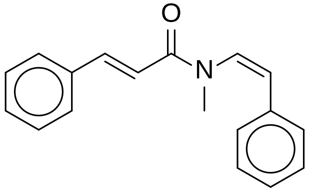	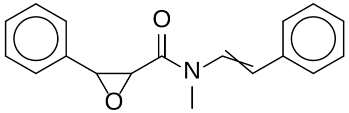	Oxidation	*Clausena lansium*	Phytochemistry, 28(2), 621-622<br>Chin. Pharm. J. (Taipei), 48(5), 367-373

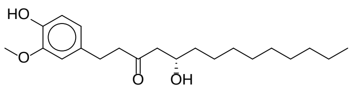	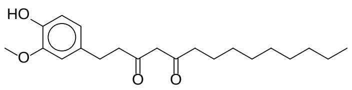	Oxidation	*Zingiber officinale*	AFS, Advances in Food Sciences, 25(1), 2-7<br>Phytotherapy Research, 17(8), 897-902
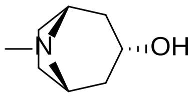	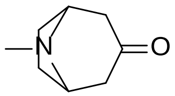	Oxidation	*Datura stramonium*	Biochemical Systematics and Ecology, 33(10), 1017-1029
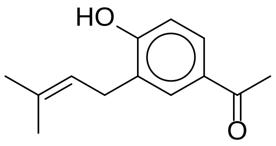	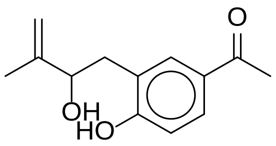	Oxidation	*helichrysum italicum*	Journal of Natural Products, 64(10), 1360-1362<br>Phytochemistry, 29(4), 1093-1095
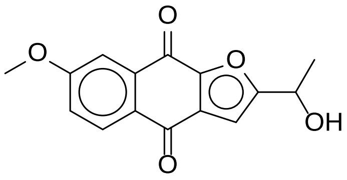	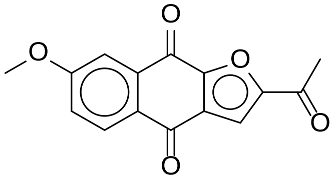	Oxidation	*Tabebuia ochracea neochrysanta*	J. Nat. Prod., 59(4), 423-424
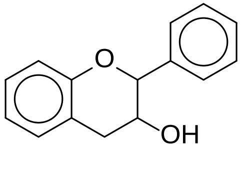	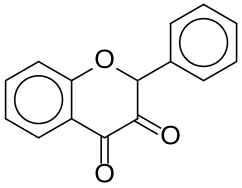	Oxidation	*Malus pumila*	Acta Horticulturae, 814(Proceedings of the XIIth Eucarpia Symposium on Fruit Breeding and Genetics, 2007, Volume 2), 545-549
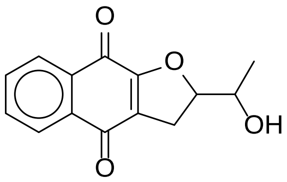	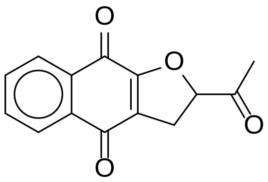	Oxidation	*Kigelia pinnata*	Phytochemistry, 32(4), 1015-1018

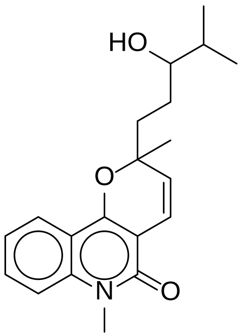	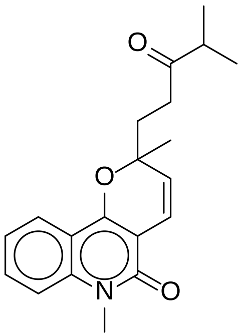	Oxidation	*Zanthoxylum simulans*	Phytochemistry, 42(1), 217-219<br>Phytochemistry, 46(3), 525-529<br>J. Nat. Prod., 57(9), 1206-1211
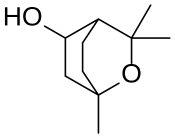	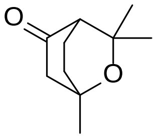	Oxidation	*Thymus vulgaris*	Flavour Fragrance J., 13(4), 259-262
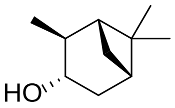	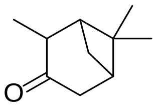	Oxidation	*Hyssopus officinalis*	Dev. Food Sci., 18(Flavors Fragrances), 171-176<br>Parfuem. Kosmet., 67(2), 116, 118
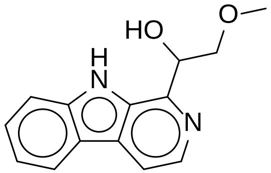	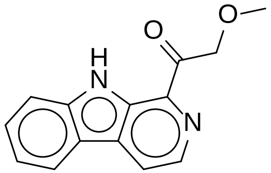	Oxidation	*Arenaria kansuensis*	Chem. Pharm. Bull., 37(7), 1808-1809
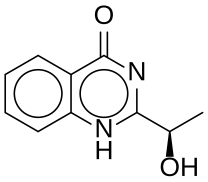	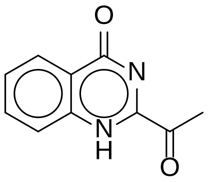	Oxidation	*Fusarium lateritium*	Can. J. Chem., 71(9), 1362-1367

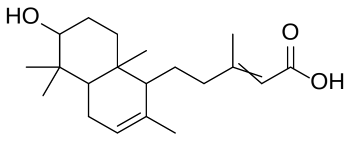	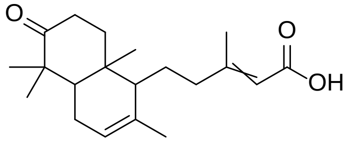	Oxidation	*Leyssera gnaphalodes*	Phytochemistry, 30(1), 211-2113
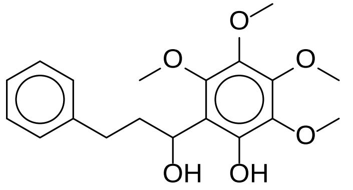	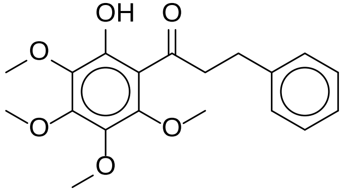	Oxidation	*Lindera lucida*	Phytochemistry, 49(7), 2141-2143
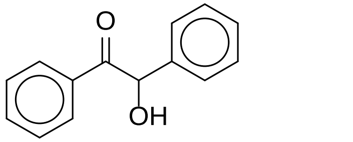	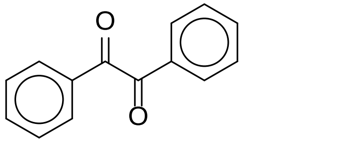	Oxidation	*Dionysia diapensifolia*	Journal of Essential Oil Research, 22(5), 386-388
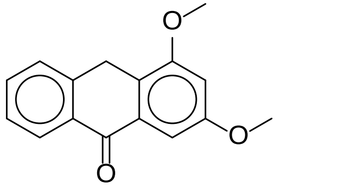	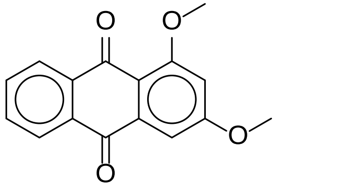	Oxidation	*Morinda citrifolia*	Natural Product Research, Part A: Structure and Synthesis, 22(13), 1128-1136<br>Archives of Pharmacal Research, 30(8), 919-923
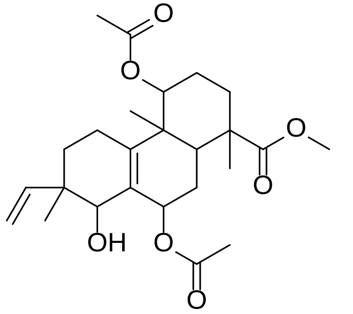	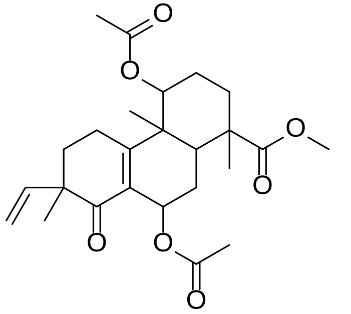	Oxidation	*Lycopus europaeus*	J. Nat. Prod., 63(3), 419-421

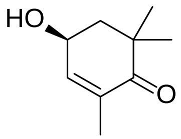	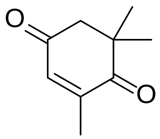	Oxidation	*Crocus sativus*	Chemical & Pharmaceutical Bulletin, 50(10), 1305-1309<br>Journal of Chromatography, A, 1216(33), 6088-6097<br>Zhongguo Xiandai Zhongyao, 10(5) , 15-17, 46
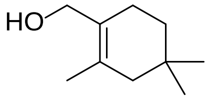	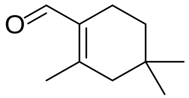	Oxidation	*Peucedanum paniculatum*	Phytochemistry (Elsevier), 66(16), 1956-1962
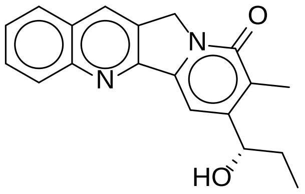	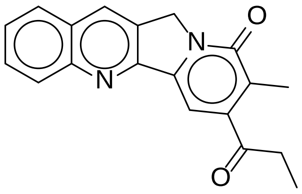	Oxidation	*Nothapodytes foetida*	Phytochemistry (Elsevier), 62(3), 461-470<br>Phytochemistry, 42(3), 907-908
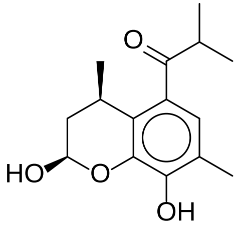	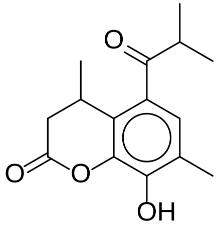	Oxidation	*Thespesia populnea*	Journal of Natural Products, 71(7), 1173-1177
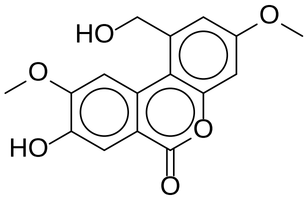	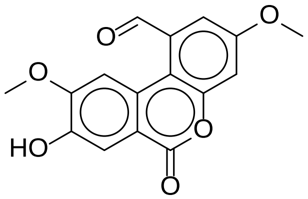	Oxidation	*Herpetospermum caudigerum*	Chemical & Pharmaceutical Bulletin, 56(2), 192-193

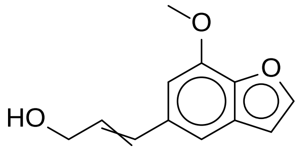	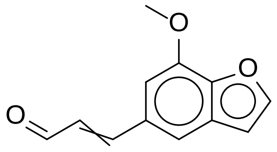	Oxidation	*Zanthoxylum wutaiense*	Journal of Natural Products, 71(7), 1146-1151
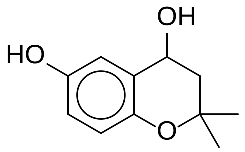	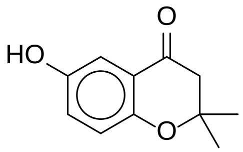	Oxidation	*Gynura elliptica*	Phytochemistry, 53(8), 833-836
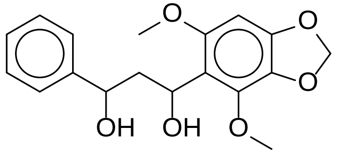	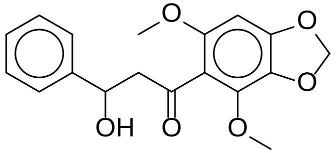	Oxidation	*helichrysum sutherlandii*	Phytochemistry, 17(11), 1935-1937
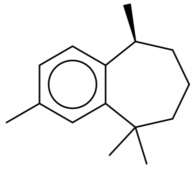	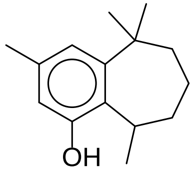	Solvolysis	*Cedrus libani*	Fitoterapia, 78(4), 323-326<br>Asian Journal of Chemistry, 17(4), 2300-2306<br>Asian Journal of Chemistry, 21(4), 2684-2694
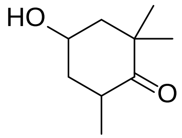	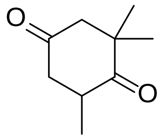	Oxidation	*porphyra tenera*	Dev. Food Sci., 10(Prog. Flavour Res.), 281-300
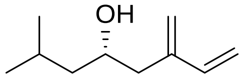	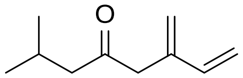	Oxidation	*Lippia multiflora*	Flavour and Fragrance Journal, 20(1), 34-38<br>Phytochemistry, 29(2), 521-522<br>J. Essent. Oil Res., 6(6), 623-630

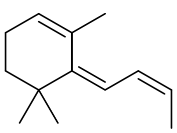	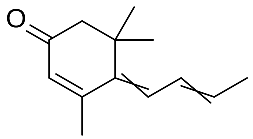	Oxidation	*Averrhoa carambola*	Guoshu Xuebao, 25(1), 119-121<br>Flavour Fragrance J., 7(4), 179-185
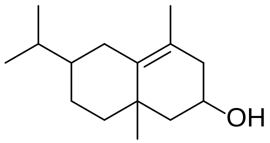	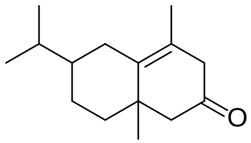	Oxidation	*Atractylodes macrocephala*	Zhongcaoyao, 35(1), 5-8
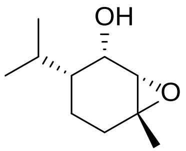	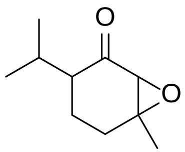	Oxidation	*Minthostachys tomentosa*	Tetrahedron: Asymmetry, 12(4), 677-683<br>Bol. Soc. Quim. Peru, 64(1), 69-73
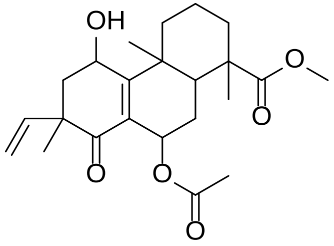	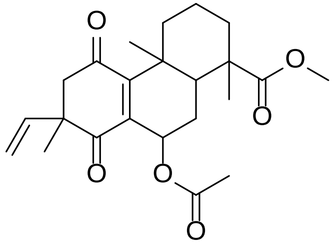	Oxidation	*Lycopus europaeus*	J. Nat. Prod., 63(3), 419-421
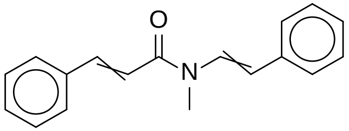	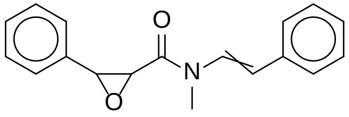	Oxidation	*Clausena lansium*	Chin. Pharm. J. (Taipei), 48(5), 367-373<br>Zhongguo Tiaoweipin, (11), 62-65

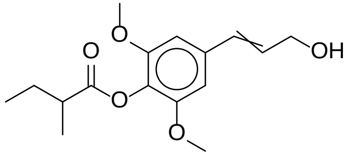	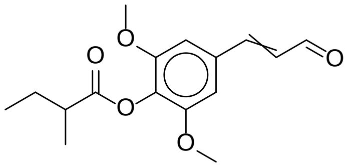	Oxidation	*Heteroplexis microcephala*	Journal of Natural Products, 72(6), 1184-1190
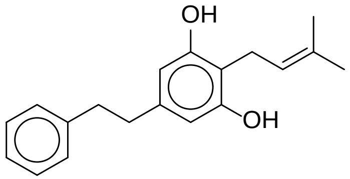	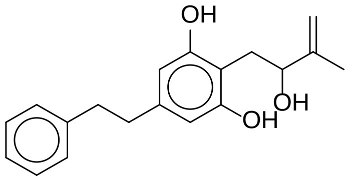	Oxidation	*Glycyrrhiza lepidota*	Phytochemistry, 22(2), 573-576<br>Phytochemistry, 28(12), 3556-3557
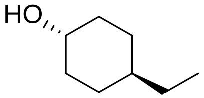	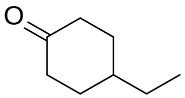	Oxidation	*Nemipterus virgatus*	Shipin Kexue (Beijing, China), 30(18), 278-281
