# Supplementary material for: Virtual Screening for Reactive Natural Products and Their Probable Artifacts of Solvolysis and Oxidation
Source: Biomolecules. 2020 Oct 27;10(11):1486. doi: 10.3390/biom10111486 (PMC7692644; doi:10.3390/biom10111486)
Supplement: Supplementary file 1 [file biomolecules-10-01486-s001.zip › 1.Example of a set of relational data.rtf]

Name:	2-(1,2-dimethoxyphenanthro[2,3-d][1,3]dioxol-4-yl)-N,N-dimethylethanamine
SMILES:	COc1cc(CCN(C)C)c2ccc3cc4c(cc3c2c1OC)OCO4
InchiKey:	DDCILWXYWBKXKC-UHFFFAOYSA-N
MF:	C21H23NO4
MW:	353
ClogP:	3.843
References: Phytochemistry 1990,29,1895-7.

Name:	(7aS)-4,9,10,11-tetramethoxy-7-methyl-6,7,7a,8-tetrahydro-5H-[1,3]benzodioxolo[6,5,4-de]benzo[g]quinoline
SMILES:	COc1cc2c(c(OC)c1OC)C[C@H]1c3c(c(OC)c4c(c3-2)OCO4)CCN1C
InchiKey:	SFHWHWVEDBDXLV-AWEZNQCLSA-N
MF:	C22H25NO6
MW:	399
ClogP:	3.2018
References: Phytochemistry 1990,29,1895-7.

Name:	Berberine
SMILES:	COc1ccc2cc3[n+](cc2c1OC)CCc1cc2c(cc1-3)OCO2
InchiKey:	YBHILYKTIRIUTE-UHFFFAOYSA-N
MF:	C20H18NO4
MW:	336
ClogP:	3.0963
References: Tianran Chanwu Yanjiu Yu Kaifa 2002,14,24-25.
Acta Botanica Sinica 2003,45,500-502.
Zhongcaoyao 1987,18,2-4.

Name:	4-(4-methoxyphenyl)but-3-en-2-one
SMILES:	COc1ccc(C=CC(C)=O)cc1
InchiKey:	WRRZKDVBPZBNJN-UHFFFAOYSA-N
MF:	C11H12O2
MW:	176
ClogP:	2.2974
References: Tianran Chanwu Yanjiu Yu Kaifa 2002,14,24-25.
Acta Botanica Sinica 2003,45,500-502.

Name:	6,7-dimethoxy-2-methylisoquinolin-1(2H)-one
SMILES:	COc1cc2ccn(C)c(=O)c2cc1OC
InchiKey:	HCHLTWGUUUDWFP-UHFFFAOYSA-N
MF:	C12H13NO3
MW:	219
ClogP:	1.5557
References: Tianran Chanwu Yanjiu Yu Kaifa 2002,14,24-25.
Acta Botanica Sinica 2003,45,500-502.

Name:	5,6,7-trimethoxy-2-methylisoquinolin-1(2H)-one
SMILES:	COc1cc2c(=O)n(C)ccc2c(OC)c1OC
InchiKey:	XFRIKMYFPGKPAP-UHFFFAOYSA-N
MF:	C13H15NO4
MW:	249
ClogP:	1.5643
References: Tianran Chanwu Yanjiu Yu Kaifa 2002,14,24-25.
Acta Botanica Sinica 2003,45,500-502.

Name:	isocorydine
SMILES:	COc1ccc2c(c1O)-c1c(OC)c(OC)cc3c1[C@H](C2)N(C)CC3
InchiKey:	QELDJEKNFOQJOY-ZDUSSCGKSA-N
MF:	C20H23NO4
MW:	341
ClogP:	3.1701
References: Phytochemistry 1990,29,1895-7.

Name:	
SMILES:	
InchiKey:	
MF:	C21H23NO5
MW:	369
ClogP:	
References: Zhongcaoyao 1987,18,2-4.

Name:	6,7-dimethoxy-3,4-dihydroisoquinolin-1(2H)-one
SMILES:	COc1cc2c(cc1OC)C(=O)NCC2
InchiKey:	MQKFSXLBPPCAGR-UHFFFAOYSA-N
MF:	C11H13NO3
MW:	207
ClogP:	0.9897
References: Tianran Chanwu Yanjiu Yu Kaifa 2002,14,24-25.
Acta Botanica Sinica 2003,45,500-502.

Name:	(6aS)-1,11-dihydroxy-2,10-dimethoxy-6,6-dimethyl-5,6,6a,7-tetrahydro-4H-dibenzo[de,g]quinolinium
SMILES:	COc1ccc2c(c1O)-c1c(O)c(OC)cc3c1[C@H](C2)[N+](C)(C)CC3
InchiKey:	YLRXAIKMLINXQY-ZDUSSCGKSA-O
MF:	C20H24NO4
MW:	342
ClogP:	3.0117
References: Phytochemistry 1990,29,1895-7.

Name:	(7aS)-4,10,11-trimethoxy-7-methyl-6,7,7a,8-tetrahydro-5H-[1,3]benzodioxolo[6,5,4-de]benzo[g]quinoline
SMILES:	COc1cc2c(cc1OC)-c1c3c(c(OC)c4c1[C@H](C2)N(C)CC4)OCO3
InchiKey:	XEZKWYLHAOYOCL-AWEZNQCLSA-N
MF:	C21H23NO5
MW:	369
ClogP:	3.1932
References: Phytochemistry 1990,29,1895-7.

Name:	6,7-dimethoxy-2-methyl-3,4-dihydroisoquinolin-1(2H)-one
SMILES:	COc1cc2c(cc1OC)C(=O)N(C)CC2
InchiKey:	BDIZBBGNYDRCCA-UHFFFAOYSA-N
MF:	C12H15NO3
MW:	221
ClogP:	1.3319
References: Acta Botanica Sinica 2003,45,500-502.

Name:	7,8-dihydro-[1,3]dioxolo[4,5-g]isoquinolin-5(6H)-one
SMILES:	O=C1NCCc2cc3c(cc21)OCO3
InchiKey:	VSOJKDUWYQCWFM-UHFFFAOYSA-N
MF:	C10H9NO3
MW:	191
ClogP:	0.7012
References: Tianran Chanwu Yanjiu Yu Kaifa 2002,14,24-25.
Acta Botanica Sinica 2003,45,500-502.

Name:	6-methyl-5,7,8,15-tetrahydrobis[1,3]benzodioxolo[5,6-c:5',6'-g]azecin-14(6H)-one
SMILES:	CN1CCc2cc3c(cc2C(=O)Cc2cc4c(cc2C1)OCO4)OCO3
InchiKey:	ZAALQOFZFANFTF-UHFFFAOYSA-N
MF:	C20H19NO5
MW:	353
ClogP:	2.5573
References: Zhongcaoyao 1987,18,2-4.

Name:	
SMILES:	COc1cc2c(cc1OC)[C@H]1[C@@H](C)c3ccc(OC)c(OC)c3C[N+]1(C)CC2.[Cl-]
InchiKey:	GDOZHUQGZPJFOC-PXUDOWQFSA-M
MF:	C23H30NO4.Cl
MW:	419.5
ClogP:	1.0861
References: Tianran Chanwu Yanjiu Yu Kaifa 2002,14,24-25.

Name:	5-hydroxy-6,7-dimethoxy-2-methyl-3,4-dihydroisoquinolin-1(2H)-one
SMILES:	COc1cc2c(c(O)c1OC)CCN(C)C2=O
InchiKey:	YIZHREJONWDRBE-UHFFFAOYSA-N
MF:	C12H15NO4
MW:	237
ClogP:	1.0375
References: Acta Botanica Sinica 2003,45,500-502.

Name:	6-hydroxy-5,7-dimethoxy-1-(4-methoxybenzyl)-2-methylisoquinolinium
SMILES:	COc1ccc(Cc2c3cc(OC)c(O)c(OC)c3cc[n+]2C)cc1
InchiKey:	LHODGQZMYZJNLP-UHFFFAOYSA-O
MF:	C20H22NO4
MW:	340
ClogP:	2.9865
References: Acta Botanica Sinica 2003,45,500-502.
